# Comparing different techniques of uterine artery occlusion during laparoscopic myomectomy: a randomized controlled trial

**DOI:** 10.1186/s12905-026-04799-z

**Published:** 2026-08-25

**Authors:** Ahmed Shoukry, Omar Ashraf Aboali, Manar Fayez Abdelquader, Ahmed M.H. Gouda

**Affiliations:** 1 Department of Obstetrics and Gynecology, Faculty of Medicine, University of Alexandria, Alexandria, Egypt; 2 Madina Women’s Hospital, Alexandria, Egypt; 3 Egyptian Ministry of Health, Alexandria, Egypt

**Keywords:** Laparoscopic myomectomy, Uterine artery occlusion, Uterine fibroids, Ovarian reserve, Estimated blood loss

## Abstract

**Background:**

Laparoscopic myomectomy carries a risk of significant intraoperative bleeding. Although uterine artery occlusion is used to reduce blood loss, its overall benefit compared with no occlusion and the relative performance of different occlusion techniques remain uncertain. This study primarily compared uterine artery occlusion, performed using different techniques, versus no occlusion during laparoscopic myomectomy, and secondarily explored the feasibility, safety, operative outcomes, and short-term ovarian reserve outcomes of temporary clipping, shoelace suture, and permanent bipolar coagulation.

**Methods:**

This prospective, single-blind, four-arm randomized controlled trial was conducted in Egypt. Of 61 randomized participants with symptomatic uterine leiomyomas, 60 received their allocated intervention and were included in the modified intention-to-treat analysis. Participants were assigned using an intended 1:1:1:1 allocation ratio. The final analyzed groups were uterine artery clipping (*n* = 15), shoelace suture (*n* = 15), bipolar coagulation (*n* = 16), and no occlusion (control; *n* = 14). The prespecified primary analysis compared uterine artery occlusion performed using different techniques versus no occlusion for estimated intraoperative blood loss and 24-hour haemoglobin decline. Secondary analyses included exploratory four-group comparisons of estimated blood loss, haemoglobin decline, operative time, and transfusion requirement, together with postoperative ovarian reserve markers—anti-Müllerian hormone and antral follicle count—assessed three months after surgery.

**Results:**

In the prespecified primary comparison, uterine artery occlusion performed using different techniques did not produce a detectable reduction in estimated intraoperative blood loss compared with no occlusion (median 260 mL [IQR 160–400] vs. 205 mL [IQR 190–300]; Hodges–Lehmann estimated difference 30 mL, 95% CI − 50 to 110; *p* = 0.416). Mean 24-hour haemoglobin decline was also similar between groups (1.17 ± 0.78 vs. 1.11 ± 0.44 g/dL; *p* = 0.759; Cohen’s d = 0.07). Operative time was longer with uterine artery occlusion (median 160 vs. 120 min; *p* = 0.007). Blood transfusion was required in 11 of 46 participants (23.9%) in the occlusion group and none of 14 participants in the no-occlusion group (risk difference 23.9% points, 95% CI 0.2–37.9; Fisher’s exact *p* = 0.053). In secondary exploratory four-group analyses, estimated blood loss differed among techniques. Bipolar coagulation had the lowest observed median blood loss and the shortest operative time among the occlusion techniques; however, superiority over no occlusion was not established. At three months, no detectable between-group differences in postoperative anti-Müllerian hormone or antral follicle count were detected after adjustment for the corresponding baseline values and age.

**Conclusions:**

In this trial, uterine artery occlusion performed using different techniques did not produce a detectable reduction in estimated intraoperative blood loss or 24-hour haemoglobin decline compared with no occlusion and was associated with longer operative time. Transfusions occurred only in the occlusion group, although the pooled comparison did not reach statistical significance. Because the observed variability in blood loss substantially exceeded the a *priori* assumption, the primary comparison was underpowered and remained compatible with clinically important benefit or harm. The exploratory technique-specific findings were method-dependent and did not establish the superiority of any individual occlusion technique. No detectable short-term between-group difference in ovarian reserve markers was observed after adjustment for baseline imbalances.

**Trial registration:**

ClinicalTrials.gov Identifier: NCT07352761. Study first submitted: 21 December 2025; study first posted: 20 January 2026. The trial was retrospectively registered.

**Supplementary Information:**

The online version contains supplementary material available at 10.1186/s12905-026-04799-z.

## Introduction

Uterine leiomyomas are the most common benign tumors of the female genital tract, affecting 40% and up to 70% of women by ages 35 and 50 years, respectively [1]. Myomas can significantly decrease quality of life because they may cause symptoms including menorrhagia, dysmenorrhea, pelvic pain, and pressure symptoms. Large myomas can also stimulate urinary tract compression, causing increased urinary frequency and urgency [2].

Several medical treatments have been successfully advocated to tackle fibroids but with no evidence of long-term effectiveness. Therefore, surgical intervention is often required especially to control the symptoms or for fertility purposes [3].

Conservative surgical management for women includes myomectomy, performed either laparoscopically or through an open approach. The laparoscopic approach is associated with less postoperative discomfort, a shorter hospital stay, and a quicker recovery [4].

Despite advances in minimally invasive surgery, conservative surgical treatment of leiomyomas still faces two major problems: the morbidity associated with surgery and the risk of recurrence of leiomyomas and/or symptoms. As regards the morbidity related to surgery, myomectomy is associated with a high risk of intra- or postoperative bleeding, requiring a blood transfusion and/or haemostatic hysterectomy in some cases [5].

To address bleeding during laparoscopic myomectomy, laparoscopic bilateral occlusion of the uterine vessels was introduced [6]. Various occlusion modalities are available, including temporary occlusion with an endoscopic vascular clip, the use of a shoelace knot with sutures, and permanent coagulation and sealing of the uterine vessels with vessel-sealing devices [7].

Consequently, the primary objective of this study was to determine whether uterine artery occlusion, performed using different techniques, reduces estimated intraoperative blood loss and 24-hour haemoglobin decline compared with no occlusion during laparoscopic myomectomy. Secondary exploratory objectives were to compare the feasibility, safety, operative outcomes, and short-term ovarian reserve outcomes of temporary clipping, shoelace suture, and permanent bipolar coagulation.

## Methods

### Study design

This was a prospective, randomized, controlled, single-blind, four-arm clinical trial with an intended 1:1:1:1 allocation ratio.

### Patient and public involvement

Patients and the public were not involved in the design, conduct, reporting, or dissemination planning of this trial.

### Setting and participants

The study was conducted at two gynecologic endoscopy centers in Alexandria, Egypt: ElShatby University Hospital (tertiary care) and Al-Madina Women Hospital (specialized obstetrics and gynecology center). Participant enrollment commenced on 1 May 2025 and was completed on 30 September 2025. Follow-up was completed on 25 February 2026.

Women with symptomatic uterine leiomyomas scheduled for laparoscopic myomectomy were screened for eligibility by the main surgeons. Inclusion criteria were fibroids measuring 3–10 cm in diameter, with a maximum of three lesions, and associated symptoms (abnormal uterine bleeding, infertility, or pressure symptoms).

Exclusion criteria comprised contraindications to laparoscopy or pneumoperitoneum, body mass index > 30 kg/m², prior midline laparotomy, previous ovarian surgery, hormonal therapy within 3 months, and clinical scenarios favoring hysterectomy (≥ 4 fibroids, adenomyosis, or recurrent disease). Patients with suspected endometrial malignancy or leiomyosarcoma on imaging, or those requiring hysteroscopic resection of submucous fibroids, were excluded according to previous literature [8, 9].

### Randomization and blinding

Participants were randomized in an intended 1:1:1:1 allocation ratio one day before surgery using a computer-generated simple randomization sequence generated in SPSS version 27 (IBM Corp., Armonk, NY, USA) by an independent statistician who was not involved in patient enrollment, perioperative care, or surgery. The study planned approximately 15 participants per randomized group, corresponding to an intended pooled allocation of 45 participants undergoing uterine artery occlusion and 15 undergoing no occlusion.

Block randomization was not used, and randomization was not stratified by center because both centers followed identical surgical protocols and the procedures were performed by the same two surgeons. Allocation concealment was maintained using sequentially numbered, opaque, sealed envelopes prepared by the statistician and opened in sequence by an independent research assistant after eligibility confirmation and written informed consent. Participants were blinded to group assignment; surgeons were not blinded because of the nature of the interventions.

One participant allocated to the no-occlusion group was withdrawn before intervention after repeat transvaginal ultrasound on the day of surgery identified a type 1 submucous fibroid component requiring hysteroscopic resection, which met a prespecified exclusion criterion. This participant did not enter the operating room, received no study intervention, and had no outcome data. To maintain the planned treated sample size, one additional eligible participant was subsequently randomized and assigned using the next sequentially numbered, opaque, sealed envelope from the original randomization sequence; this participant was allocated to the coagulation group. Because envelopes were opened only once and in sequence, allocation concealment for subsequent participants was not altered. No intraoperative crossovers occurred. The final analyzed group sizes were: clipping, *n* = 15; shoelace suture, *n* = 15; coagulation, *n* = 16; and no occlusion, *n* = 14, corresponding to an achieved pooled allocation of 46:14.

## Interventions

### Participants were allocated to one of the following four groups

- Group I (Clipping): Laparoscopic myomectomy with temporary bilateral uterine artery occlusion using laparoscopic vascular clips (LigaV^®^ titanium clips, Grena Ltd., UK), removed after myometrial closure.
- Group II (Shoelace suture): Laparoscopic myomectomy with temporary bilateral uterine artery occlusion using a removable shoelace suture technique (Vicryl 1 − 0, polyglactin 910).
- Group III (Coagulation): Laparoscopic myomectomy with permanent bilateral uterine artery occlusion using bipolar coagulation (Karl Storz, Tuttlingen, Germany).
- Group IV (Control): Laparoscopic myomectomy without uterine artery occlusion.

All procedures were performed by two experienced laparoscopic surgeons using a standardized surgical technique. Concurrent care and perioperative management were identical across all four groups.

### Surgical technique

All procedures were performed laparoscopically under general anesthesia with the patient in the dorsal lithotomy position using Allen stirrups. Pneumoperitoneum was established via Veress needle, and the primary trocar was inserted through the umbilicus or Palmer’s point, depending on uterine size. The operating table was adjusted to a 30° Trendelenburg position, and intra-abdominal pressure was maintained at 13–14 mmHg. A 10-mm camera port was placed, followed by insertion of two 5-mm ancillary trocars in the iliac fossae and an additional suprapubic port. A uterine manipulator was used in all suitable patients to facilitate uterine mobilization.

### Retroperitoneal dissection and uterine artery identification

In patients assigned to uterine artery occlusion groups (Groups I–III), retroperitoneal access was obtained prior to myomectomy. A 2–3 cm vertical peritoneal incision was made to enter the retroperitoneal space after identifying the anatomical triangle (Supplementary Fig. 1) bounded by the round ligament anteriorly, the infundibulopelvic ligament posteriorly, and the external iliac artery laterally [10, 11]. Upon entering this avascular space, careful blunt dissection was performed to identify the ureter and the internal iliac artery. The ureter was recognized by its characteristic peristalsis and gently mobilized medially using atraumatic graspers. The uterine artery, originating from the anterior division of the internal iliac artery and crossing over the ureter, was then identified and skeletonized bilaterally. The same steps were repeated on the contralateral side to ensure complete bilateral exposure of the uterine arteries. In selected cases, alternative anterior or posterior approaches were employed depending on fibroid size, location, and surgical accessibility [10].

### Uterine artery occlusion techniques

- Clipping: Temporary occlusion was achieved using LigaV^®^ titanium clips applied bilaterally at the level of the uterine artery origin. Clips were deployed using a dedicated laparoscopic clip applier, ensuring precise placement and secure vessel occlusion without compromising adjacent structures. Clips were removed after completion of myometrial closure to restore uterine perfusion (Supplementary Fig. 2).
- Shoelace suture: Temporary occlusion was performed using a removable shoelace knot technique, as described by Pisat et al. [12]. A Vicryl 1 − 0 suture was passed around the uterine artery at its origin, and a double-loop configuration was created to form a reversible knot (Supplementary Fig. 3). The suture was tightened to achieve effective vascular occlusion during myomectomy. Following completion of uterine repair, the knot was released by traction on one end of the suture, restoring uterine blood flow without the need for metallic devices.
- Coagulation: Permanent uterine artery occlusion was achieved using laparoscopic bipolar forceps. After isolation of the uterine artery, controlled bipolar energy was applied under direct visualization until complete desiccation of the vessel was achieved (Supplementary Fig. 4). Care was taken to avoid thermal spread to adjacent structures, particularly the ureter and utero-ovarian anastomoses.

### Myomectomy

Following vascular control (in Groups I–III), laparoscopic myomectomy was performed in a standardized manner. A vertical incision was made over the uterine serosa directly above the fibroid using monopolar or ultrasonic energy. The fibroid was grasped using a myoma screw and enucleated using traction and counter-traction along the natural cleavage plane. Haemostasis was achieved using selective bipolar coagulation of bleeding vessels. The myometrial defect was repaired in two or three layers using continuous sutures with Vicryl 0–1 to ensure adequate reconstruction and haemostasis. Extracted myomas were placed in a specimen retrieval bag and removed using contained morcellation or extraction techniques as appropriate. After myometrial closure, temporary occlusion techniques (clipping and shoelace suture) were reversed, and restoration of uterine perfusion was confirmed visually.

### Outcomes

#### Primary outcomes

- Estimated intraoperative blood loss was calculated as the total volume collected in the calibrated suction canister minus the volume of irrigation fluid used during the procedure. Irrigation volume was recorded throughout surgery by the circulating operating-room nurse, and the final EBL calculation was completed immediately after surgery using the anesthesia record and suction measurements. Because the allocated surgical technique was necessarily visible throughout the procedure, neither the operating surgeons nor the operating-room staff responsible for recording intraoperative blood loss were blinded to treatment allocation.
- Decline in haemoglobin concentration (Hb decline), defined as the difference between preoperative haemoglobin and haemoglobin measured at the scheduled 24-hour postoperative assessment.

#### Secondary outcomes

- Total operative time (minutes), defined from skin incision to skin closure.
- Duration of uterine artery occlusion (minutes; applicable to Groups I–III only).
- Ovarian reserve parameters at 3 months postoperatively: serum AMH and transvaginal ultrasound AFC.
- Intraoperative complications (including conversion to laparotomy or hysterectomy and need for technique modification).
- Postoperative complications, graded according to the Clavien-Dindo classification system [13].
- Blood transfusion requirement (incidence and number of units transfused).
  - ◦ Blood transfusion was administered according to institutional criteria: haemoglobin < 8 g/dL, haemoglobin 8–9 g/dL with clinical signs of haemodynamic instability, or perioperative haemoglobin decline > 3 g/dL. Postoperative haemoglobin was measured routinely at 24 h after surgery. In patients who received transfusion before this time point, the 24-hour haemoglobin value represented a post-transfusion measurement. For participants who received transfusion before the 24-hour measurement, the pre-transfusion haemoglobin value (measured 4–6 h postoperatively, at the time of the transfusion decision) was also recorded and used in an additional sensitivity analysis.

### Follow-up

Postoperative haemoglobin concentration was measured at 24 h after surgery. Ovarian reserve was reassessed at 3 months (± 7 days) using serum AMH and transvaginal ultrasound for AFC. The three-month ovarian reserve assessments were performed by assessors blinded to group allocation. Laboratory personnel measuring postoperative haemoglobin were also unaware of the allocated surgical technique.

### Sample size calculation

The sample size was calculated a *priori* based on the primary outcome of intraoperative estimated blood loss. Based on unpublished institutional data, the mean estimated blood loss during conventional laparoscopic myomectomy was approximately 190 mL, with a standard deviation of 60 mL. A reduction of at least 25% was considered clinically meaningful, corresponding to an absolute reduction of approximately 50 mL.

Using a two-sided alpha level of 0.05 and 80% power, a conventional two-group calculation with equal allocation yielded a minimum total sample size of 46 participants. Because the ethics-approved protocol specified a four-arm design with 1:1:1:1 randomization, the recruitment target was increased to 60 treated participants, with approximately 15 participants planned per group. This design corresponded to an intended pooled allocation of 45 participants undergoing uterine artery occlusion and 15 undergoing no occlusion for the primary comparison.

The primary pooled analysis combined the three uterine artery occlusion groups and compared them with the no-occlusion group. The four-group design also permitted exploratory comparisons among the individual techniques; however, the study was not powered for definitive superiority testing between individual techniques. Consequently, four-group and pairwise comparisons were interpreted as secondary exploratory analyses. No interim analyses or stopping guidelines were planned.

### Ethical approval and trial registration

The study protocol was approved by the Ethics Committee of the Faculty of Medicine, Alexandria University, Egypt (IRB No. 0307946; approval date: 13 October 2024). The study was conducted in accordance with the ethical principles of the Declaration of Helsinki (2013 revision), and all participants provided written informed consent before enrollment. The trial was retrospectively registered at ClinicalTrials.gov (Identifier: NCT07352761). The study was first submitted to the registry on 21 December 2025 and first posted on 20 January 2026. The original ethics-approved protocol, including the study design, eligibility criteria, four-arm 1:1:1:1 randomization plan, interventions, specified outcomes, and follow-up schedule, was finalized before recruitment commenced. No changes were made to the eligibility criteria, interventions, or specified outcomes after enrollment began. Additional adjusted and sensitivity analyses are identified transparently as such below.

### Statistical analysis

Analyses were performed using SPSS version 27. Continuous variables were assessed for normality using the Shapiro–Wilk test and are reported as mean ± standard deviation or median (interquartile range), as appropriate. Categorical variables are presented as counts and percentages.

The prespecified primary analysis compared uterine artery occlusion performed using the three different techniques combined versus no uterine artery occlusion for estimated intraoperative blood loss and 24-hour haemoglobin decline. Comparisons among the four randomized groups were considered secondary exploratory analyses.

For the primary pooled comparison, continuous outcomes were analyzed using the independent-samples t-test or Mann–Whitney U test according to data distribution, and categorical outcomes were compared using Fisher’s exact test. For secondary exploratory four-group comparisons, continuous variables were analyzed using one-way analysis of variance (ANOVA) or the Kruskal–Wallis test, as appropriate, and categorical variables were compared using Fisher’s exact test. For four-group Kruskal–Wallis analyses, post-hoc pairwise comparisons were performed using Mann–Whitney U tests with Bonferroni correction for six comparisons, giving an adjusted significance threshold of α = 0.0083. The Hodges–Lehmann estimator was used as the non-parametric point estimate of the between-group location difference, with 95% confidence intervals. For the pooled transfusion comparison, the absolute risk difference with its 95% confidence interval was calculated using Newcombe’s score method, while the corresponding p value was obtained using Fisher’s exact test.

The exploratory four-group EBL findings were further examined using adjusted models to assess the influence of baseline imbalances. Because estimated blood loss was markedly right-skewed (Shapiro–Wilk *p* < 0.001) and strictly positive, ANCOVA on log-transformed EBL was used as the principal adjusted model. Adjusted geometric means were back-transformed to the original mL scale, and geometric mean ratios with 95% confidence intervals were calculated relative to the no-occlusion group. Log-ANCOVA was selected because it accommodates right-skewed positive-valued outcomes and provides clinically interpretable back-transformed estimates. Quade’s rank-based ANCOVA and conventional (parametric) ANCOVA on raw EBL were performed as sensitivity analyses. Models progressively adjusted for age, baseline haemoglobin, fibroid count, and largest-fibroid diameter. The adjusted pairwise comparisons derived from the fully adjusted log-ANCOVA were considered exploratory and were not corrected for multiple testing.

Haemoglobin decline was initially examined using ANCOVA with adjustment for age and subsequently with adjustment for age and baseline haemoglobin. Haemoglobin decline was calculated as preoperative haemoglobin minus the scheduled 24-hour postoperative haemoglobin value; therefore, positive values indicated a postoperative reduction in haemoglobin. Because the 24-hour value represented a post-transfusion measurement in participants transfused before this assessment, a sensitivity analysis was performed using the pre-transfusion haemoglobin value for transfused participants. These values were retrieved from the source clinical records and had been measured 4–6 h postoperatively at the time of the transfusion decision. For participants who did not receive a transfusion, the scheduled 24-hour postoperative haemoglobin value was retained. The pooled sensitivity comparison was performed using Welch’s t-test because of unequal variances, and the four-group comparison was assessed using one-way ANOVA. Effect sizes for these haemoglobin sensitivity analyses are reported as Cohen’s d for the pooled comparison and partial η² for the four-group comparison.

Secondary ovarian reserve outcomes, including AMH and AFC at 3 months, were analyzed using ANCOVA models adjusted for the corresponding baseline value and age. Rank-based ANCOVA using Quade’s method was applied when model assumptions were violated. Within-group preoperative-to-postoperative changes were assessed using the Wilcoxon signed-rank test.

Effect sizes are reported as ε² for Kruskal–Wallis tests, r for Mann–Whitney U tests, Cohen’s d for t-tests, and partial η² for ANOVA and ANCOVA models, as appropriate. Epsilon-squared and partial eta-squared values were interpreted descriptively and were not categorized using r-based thresholds. All tests were two-sided. A p value < 0.05 was considered statistically significant, except for Bonferroni-adjusted pairwise comparisons, for which α = 0.0083 was used.

The primary analysis was performed in a modified intention-to-treat population, defined as all randomized participants who received their allocated intervention and had outcome data available. One participant was withdrawn before intervention, received no study procedure, and had no outcome data; therefore, this participant was not included in the outcome analysis. One additional eligible participant was subsequently randomized using the next sequentially numbered, opaque, sealed envelope from the original allocation sequence to maintain the planned treated sample size. As no intraoperative crossovers occurred among treated participants, the as-treated analysis was identical to the modified intention-to-treat analysis.

This study was reported in accordance with the CONSORT 2025 statement. The completed CONSORT 2025 checklist is provided as a supplementary file.

## Results

**Fig. 1:**
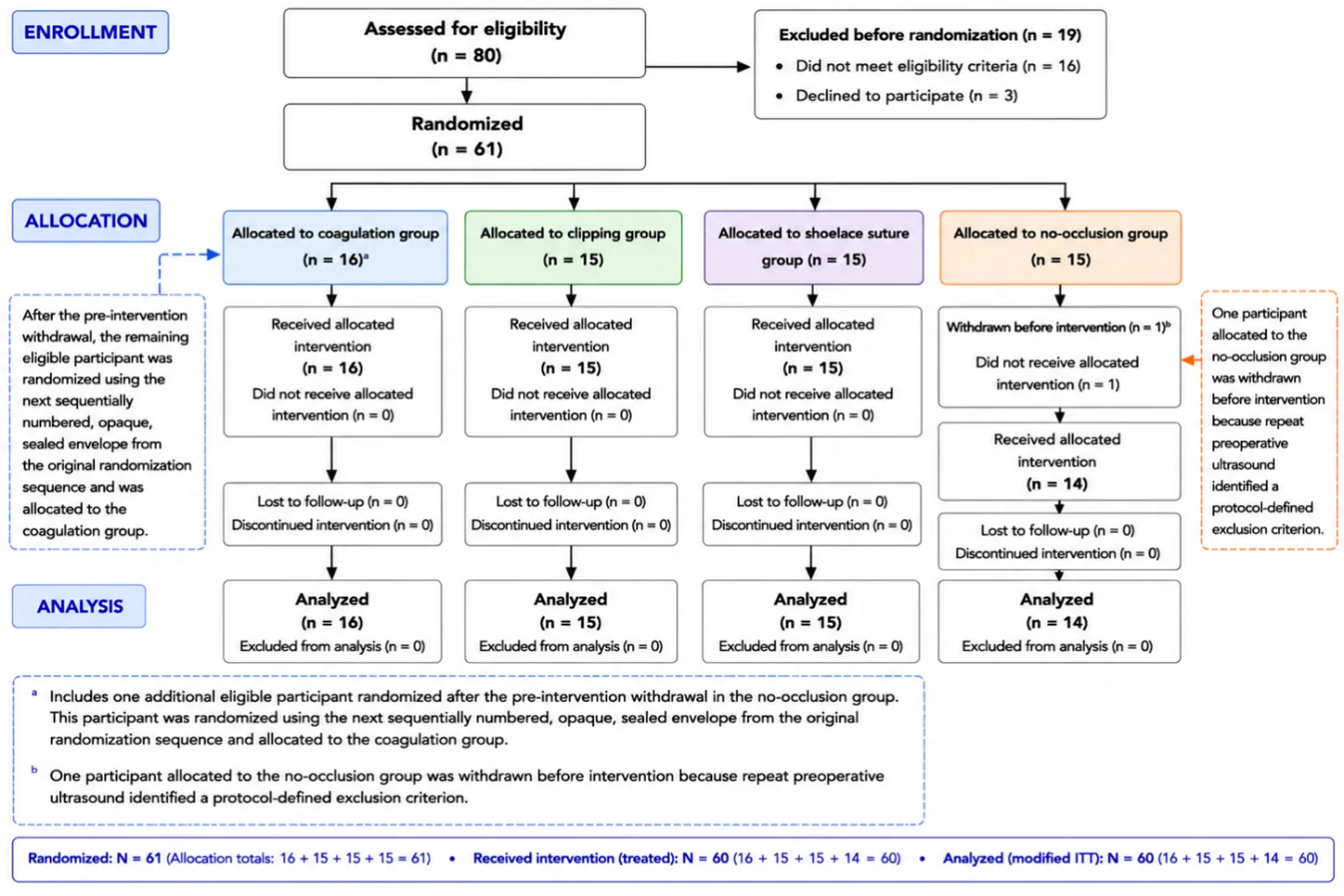
CONSORT flow diagram

Eighty women were assessed for eligibility, of whom 19 were excluded: 16 did not meet the eligibility criteria and three declined participation. Of the 61 eligible women, 60 were initially randomized. One participant allocated to the no-occlusion group was withdrawn before intervention after repeat preoperative ultrasonography identified a protocol-defined exclusion criterion. To maintain the planned treated sample size, the remaining eligible participant was subsequently randomized using the next sequentially numbered, opaque, sealed envelope from the original randomization sequence and was allocated to the coagulation group. Thus, 61 participants were randomized overall, of whom 60 received their allocated intervention and were included in the modified intention-to-treat analysis (coagulation, *n* = 16; clipping, *n* = 15; shoelace suture, *n* = 15; no occlusion, *n* = 14). Participant flow is summarized in Fig. 1.

### Baseline characteristics

Baseline characteristics are presented in Table 1. The four groups were comparable in BMI (*p* = 0.738), preoperative haemoglobin (*p* = 0.959), largest fibroid diameter (*p* = 0.213), and most presenting symptoms, including abnormal uterine bleeding, dysmenorrhea, and pelvic pain (all *p* > 0.05). However, several baseline imbalances were observed, most likely reflecting chance imbalance in this relatively small four-arm trial.

Women in the no-occlusion group were older than those in the other groups (median 40 years, IQR 38–41, compared with 33–35 years in the remaining groups; *p* = 0.010). Parity and gravidity were higher in the coagulation and no-occlusion groups than in the clipping and shoelace groups (*p* = 0.014 and *p* = 0.012, respectively). Infertility was more frequent in the clipping group (80%) than in the no-occlusion group (28.6%; *p* = 0.016). Fibroid count also differed significantly, with the clipping group having a higher number of fibroids than the other groups (*p* = 0.001).

Baseline ovarian reserve markers differed significantly across groups. Both AMH (*p* = 0.018) and AFC (*p* = 0.039) were lower in the no-occlusion and coagulation groups than in the clipping and shoelace groups. Therefore, postoperative ovarian reserve outcomes were interpreted primarily using adjusted models accounting for the corresponding baseline value and age, rather than relying solely on unadjusted postoperative comparisons or simple change scores.

**Table 1:** Baseline characteristics of study participants by randomization group

Variable	Clipping (*n* = 15)	Shoelace (*n* = 15)	Coagulation (*n* = 16)	No Occlusion (*n* = 14)	*p*-value
Demographics					
** Age (years)**,** median (Q1–Q3)**	33 (31–40)	35 (27–37)	35 (31–39)	40 (38–41)	0.01
** BMI (kg/m²)**,** median (Q1–Q3)**	28 (26–29)	28 (26–29)	28 (25–29)	27 (25–29)	0.738
** Parity**,** median (Q1–Q3)**	0 (0–1)	0 (0–1)	1.5 (0–3)	2 (0–3)	0.014
** Gravidity**,** median (Q1–Q3)**	0 (0–1)	0 (0–3)	2 (0.25–3)	2.5 (0–4)	0.012
**Presenting symptoms**					
** Infertility**,** n (%)**	12 (80%)	10 (66.7%)	6 (37.5%)	4 (28.6%)	0.016
*** Primary***	10 (66.7%)	7 (46.7%)	4 (25%)	3 (21.4%)	
*** Secondary***	2 (13.3%)	3 (20%)	2 (12.5%)	1 (7.1%)	
** Abnormal uterine bleeding**,** n (%)**	13 (86.7%)	13 (86.7%)	14 (87.5%)	10 (71.4%)	0.7
** Dysmenorrhea**,** n (%)**	7 (46.7%)	6 (40%)	11 (68.8%)	10 (71.4%)	0.23
** Pelvic pain**,** n (%)**	5 (33.3%)	6 (40%)	5 (31.3%)	9 (64.3%)	0.27
**Fibroid characteristics**					
** Fibroid count**,** median (Q1–Q3)**	2 (1–3)	1 (1–1)	1 (1–1)	1 (1–2)	0.001
** Largest fibroid diameter (cm)**,** mean ± SD**	6.8 ± 1.7	6.5 ± 1.6	6.3 ± 2.3	5.4 ± 1.5	0.213
**Preoperative laboratory values**					
** Haemoglobin (g/dL)**,** mean ± SD**	12.3 ± 0.83	12.2 ± 1.16	12.1 ± 0.68	12.2 ± 0.7	0.959
** AMH (ng/mL)**,** median (Q1–Q3)**	2.12 (1.44–3.8)	3.1 (1.2–3.9)	1.58 (1.12–2.79)	1.26 (0.88–1.69)	0.018
** AFC (total)**,** median (Q1–Q3)**	16 (10–28)	20 (10–26)	14 (12–17.75)	11 (6.75–15)	0.039

Data are presented as mean ± SD, median (Q1–Q3), or n (%), as appropriate. Continuous variables were compared using one-way ANOVA for normally distributed variables and the Kruskal–Wallis test for non-normally distributed variables. Categorical variables were compared using Fisher’s exact test. *AMH*  anti-Müllerian hormone; *AFC* antral follicle count, *BMI*  body mass index

### Prespecified primary analysis: uterine artery occlusion versus no occlusion

The prespecified primary analysis compared uterine artery occlusion performed using different techniques with no uterine artery occlusion. As shown in Table 2, participants who underwent uterine artery occlusion by clipping, shoelace suture, or coagulation were analyzed together as the intervention group (*n* = 46) and compared with the no-occlusion control group (*n* = 14). Preoperative haemoglobin was comparable between groups (12.21 ± 0.89 vs. 12.22 ± 0.71 g/dL; *p* = 0.974).

Estimated blood loss did not differ significantly between the intervention and control groups (median 260 mL, IQR 160–400 vs. 205 mL, IQR 190–300, Hodges–Lehmann estimate of between-group location difference 30 mL, 95% CI − 50 to 110; *p* = 0.416; *r* = 0.10). Blood transfusion was required in 11 of 46 participants in the intervention group (23.9%) compared with none in the control group, corresponding to a risk difference of 23.9% points (95% CI 0.2–37.9; Fisher’s exact *p* = 0.053). Haemoglobin decline was also similar between groups (1.17 ± 0.78 vs. 1.11 ± 0.44 g/dL; *p* = 0.759; Cohen’s d = 0.07).

Total operative time was longer in the intervention group than in the no-occlusion group (median 160 min, IQR 120–210 vs. 120 min, IQR 110–130; *p* = 0.007). This pooled estimate reflects the combined distribution of the clipping, shoelace suture, and coagulation groups.

Hospital stay was statistically longer in the intervention group (median 1 day, IQR 1–2 vs. 1 day, IQR 1–1; *p* = 0.011), although the median duration was identical at 1 day in both groups, limiting the clinical relevance of this difference.

Unadjusted postoperative AMH and AFC were higher in the intervention group (AMH: median 2.04 vs. 1.07 ng/mL, *p* = 0.004; AFC: median 15 vs. 9, *p* = 0.003), reflecting baseline imbalance in ovarian reserve markers rather than treatment benefit. After adjustment for the corresponding baseline ovarian reserve marker and age, these differences were no longer statistically significant (AMH: F[1,56] = 0.967, *p* = 0.330, partial η² = 0.017; AFC: F[1,56] = 0.148, *p* = 0.702, partial η² = 0.003). The magnitude of ovarian reserve decline was also similar between groups (ΔAMH: median − 0.10 vs. −0.15 ng/mL, *p* = 0.655; ΔAFC: −1 vs. −2, *p* = 0.395). These findings indicate that no detectable short-term between-group difference in ovarian reserve markers was observed after adjustment for baseline imbalance; however, they should not be interpreted as evidence of equivalence or definitive ovarian safety.

**Table 2:** Pooled intervention groups (clipping + shoelace + coagulation) versus control (no occlusion)

Outcome	Pooled Intervention (*n* = 46)	Control (*n* = 14)	*p*-value	Effect estimate
Intraoperative blood loss				
Estimated blood loss (mL), median (Q1–Q3)	260 (160–400)	205 (190–300)	0.416	*r* = 0.10 HL = 30 mL (95% CI − 50 to 110)
** Blood transfusion required**,** n (%)**	**11 (23.9%)**	**0 (0%)**	**0.053**	**RD = 23.9% (95% CI 0.2 to 37.9)**
Units transfused *	**14**	**0**	**—**	**—**
**Haemoglobin**				
** Preoperative Hb (g/dL)**,** mean ± SD**	**12.21 ± 0.89**	**12.22 ± 0.71**	**0.974**	**d = − 0.01**
** Postoperative Hb at 24 h (g/dL)**,** median (Q1–Q3)**	**11.15 (10.6–12)**	**11 (10.9–11.3)**	**0.706**	*r* = 0.04
Hb decline ** (g/dL),** mean ± SD**	**1.17 ± 0.78**	**1.11 ± 0.44**	**0.759**	**d = 0.07**
**Operative time**				
** Total operative time (min)**,** median (Q1–Q3)**	**160 (120–210)**	**120 (110–132.5)**	**0.007**	*r* = 0.34
** Hospital stay (days)**,** median (Q1–Q3)**	**1 (1–2)**	**1 (1–1)**	**0.011**	*r* = 0.25
**Ovarian reserve**				
** Postoperative AMH at 3 months (ng/mL)**,** median (Q1–Q3)**	**2.04 (1.1–3.34)**	**1.07 (0.85–1.57)**	**0.004**	*r* = 0.37
ANCOVA-adjusted group effect on postop AMH ***	**—**	**—**	**0.330**	**partial η² = 0.017**
** ΔAMH (ng/mL)**,** median (Q1–Q3)**	**−0.10 (− 0.19 to − 0.07)**	**−0.15 (− 0.2 to − 0.03)**	**0.655**	*r* = 0.05
** Postoperative AFC**,** median (Q1–Q3)**	**15 (9–24)**	**9 (5.75–12.5)**	**0.003**	*r* = 0.38
ANCOVA-adjusted group effect on postop AFC ***	**—**	**—**	**0.702**	**partial η² = 0.003**
** ΔAFC**,** median (Q1–Q3)**	**−1 (− 2 to 0)**	**−2 (− 2 to 0)**	**0.395**	*r* = 0.09

The pooled intervention group included participants allocated to temporary clipping, shoelace suture, or bipolar coagulation. Data are presented as mean ± standard deviation (SD), median (Q1–Q3), or number (%), as appropriate. Continuous variables were compared using the independent-samples t-test or Mann–Whitney U test according to data distribution, and categorical variables were compared using Fisher’s exact test. Haemoglobin decline was calculated as preoperative haemoglobin minus the 24-hour postoperative haemoglobin value; therefore, positive values indicate a postoperative reduction. In participants transfused before the 24-hour assessment, the postoperative haemoglobin value represented a post-transfusion measurement. Institutional transfusion criteria were haemoglobin < 8 g/dL, haemoglobin 8–9 g/dL with clinical signs of haemodynamic instability, or a perioperative haemoglobin decline > 3 g/dL. Units transfused are reported descriptively. The Hodges–Lehmann estimate represents the non-parametric between-group location difference, calculated as uterine artery occlusion minus no occlusion; positive values indicate higher estimated blood loss with occlusion. The transfusion risk difference was calculated as the risk in the uterine artery occlusion group minus the risk in the no-occlusion group, with its 95% confidence interval calculated using Newcombe’s score method; the corresponding *p* value was obtained using Fisher’s exact test. ANCOVA models for postoperative AMH and AFC were adjusted for the corresponding baseline value and age. Effect sizes are reported as r for Mann–Whitney U tests, Cohen’s d for independent-samples t-tests, and partial η² for ANCOVA models. *Hb* haemoglobin; *AMH* anti-Müllerian hormone, *AFC* antral follicle count, *ANCOVA* analysis of covariance, *CI* confidence interval, *HL* Hodges–Lehmann, *RD* risk difference

### Secondary exploratory four-group comparison: intraoperative blood loss and haemoglobin decline

As a secondary exploratory analysis, the primary outcomes were also examined across the four randomized groups to explore potential differences among individual uterine artery occlusion techniques (Table 3). Estimated blood loss differed across groups (Kruskal–Wallis H [3] = 10.998, *p* = 0.012; ε² = 0.186, 95% CI 0.08–0.32). The lowest observed median blood loss was recorded in the coagulation group (150 mL, IQR 120–300), whereas the highest was observed in the clipping group (360 mL, IQR 250–450). The ε² value was interpreted descriptively, suggesting that group allocation was associated with variation in ranked estimated blood loss in this exploratory analysis. However, this finding should not be interpreted as definitive evidence of a technique effect, because the analysis was underpowered for pairwise comparisons and was potentially confounded by baseline imbalance in fibroid burden, particularly fibroid count and largest fibroid diameter, as further explored in the sensitivity analyses below.

**Table 3:** Four-group comparison: intraoperative blood loss and haemoglobin decline

Outcome	Clipping (*n* = 15)	Shoelace (*n* = 15)	Coagulation (*n* = 16)	No Occlusion (*n* = 14)	*p*-value	Effect Size (95% CI)
Intraoperative blood loss						
** Estimated blood loss (mL)**,** median (Q1–Q3)**	360 (250–450)	250 (180–400)	150 (120–300)	205 (190–300)	0.012	ε² = 0.186 (0.08–0.32)
** Blood transfusion required**,** n (%)**	5 (33.3%)	3 (20%)	3 (18.8%)	0 (0%)	0.140	
Units transfused *	6	4	4	0		
**Haemoglobin**						
** Preoperative Hb (g/dL)**,** mean ± SD**	12.3 ± 0.83	12.2 ± 1.16	12.1 ± 0.68	12.2 ± 0.7	0.959	
** Postoperative Hb at 24 h (g/dL)**,** median (Q1–Q3)**	11.2 (10.1–12.1)	11.1 (10–12)	11.2 (10.7–11.5)	11 (10.9–11.3)	0.900	
Hb decline ** (g/dL),** mean ± SD**	1.30 ± 1.01	1.01 ± 0.69	1.18 ± 0.61	1.11 ± 0.44	0.715	partial η² = 0.025 (0–0.12)

Data are presented as mean ± SD, median (Q1–Q3), or n (%), as appropriate. This table presents secondary exploratory four-group comparisons

Estimated blood loss and postoperative Hb were compared using the Kruskal–Wallis test; preoperative Hb and Hb decline were compared using one-way ANOVA; categorical variables were compared using Fisher’s exact test. Hb decline was calculated as preoperative Hb minus 24-hour postoperative Hb; therefore, positive values indicate postoperative haemoglobin reduction

In participants transfused before the 24-hour measurement, postoperative Hb represents a post-transfusion value. Transfusion criteria were Hb < 8 g/dL, Hb 8–9 g/dL with clinical signs of haemodynamic instability, or perioperative Hb decline > 3 g/dL. Units transfused are reported descriptively

Effect sizes are reported as ε² for Kruskal–Wallis tests and partial η² for ANOVA models

*EBL*  estimated blood loss, *Hb* haemoglobin

Pairwise comparisons were performed using Mann–Whitney U tests with Bonferroni correction for six comparisons, giving an adjusted significance threshold of α = 0.0083. The Hodges–Lehmann estimator was used as the point estimate of the median between-group difference, with 95% confidence intervals. Estimated blood loss was significantly lower in the coagulation group than in the clipping group, corresponding to a Hodges–Lehmann median difference of 140 mL for clipping versus coagulation (95% CI 50–240; simple difference of medians 210 mL; exact *p* = 0.0049). The comparison between clipping and no occlusion did not meet the Bonferroni-adjusted significance threshold despite a simple median difference of 155 mL (Hodges–Lehmann estimate 110 mL, 95% CI 40–200; exact *p* = 0.0093). No other pairwise comparison met the adjusted significance threshold (Table 4).

**Table 4:** Post-hoc pairwise comparisons for estimated blood loss

Comparison	Simple median difference (mL)	HL estimate (mL)	95% CI for Difference	Exact *p*-value	Significant at α = 0.0083
**Clipping vs. Shoelace**	110	70	−20 to 160	0.135	No
**Clipping vs. Coagulation**	210	140	50 to 240	0.0049	Yes
**Clipping vs. No occlusion**	155	110	40 to 200	0.0093	No
**Shoelace vs. Coagulation**	100	70	−20 to 160	0.132	No
**Shoelace vs. No occlusion**	45	20	−50 to 120	0.406	No
**Coagulation vs. No occlusion**	-55	−50	−90 to 50	0.175	No

Pairwise comparisons were performed using Mann–Whitney U tests. Bonferroni correction was applied for six comparisons, giving an adjusted significance threshold of α = 0.0083. Exact unadjusted p-values are reported, and statistical significance was judged against the Bonferroni-adjusted threshold. “Simple median difference” represents the difference between observed group medians, calculated as group 1 minus group 2 The Hodges–Lehmann estimate represents the non-parametric estimate of the between-group location difference, with 95% confidence intervals., with 95% confidence intervals. *EBL* estimated blood loss

Blood transfusion was required in 11 participants overall (18.3%). The transfusion rate was highest in the clipping group (5/15, 33.3%), followed by the shoelace group (3/15, 20.0%), the coagulation group (3/16, 18.8%), and the no-occlusion group (0/14, 0%). This difference was not statistically significant (Fisher’s exact test, *p* = 0.140).

Despite the observed difference in estimated blood loss, postoperative haemoglobin concentration at 24 h did not differ significantly across groups (Kruskal–Wallis *p* = 0.900). Haemoglobin decline also did not differ significantly (one-way ANOVA, F[3,56] = 0.455, *p* = 0.715; partial η² = 0.025, 95% CI 0–0.12).

Because the scheduled 24-hour haemoglobin value was obtained after transfusion in 11 participants, a sensitivity analysis was performed using their pre-transfusion haemoglobin values, which were retrieved from the source clinical records and had been measured 4–6 h postoperatively at the time of the transfusion decision. For non-transfused participants, the scheduled 24-hour value was retained. In this sensitivity analysis, mean haemoglobin decline was 1.43 ± 1.04 g/dL in the uterine artery occlusion group and 1.11 ± 0.44 g/dL in the no-occlusion group, corresponding to a mean difference of 0.32 g/dL (Welch’s t-test *p* = 0.106; Cohen’s d = 0.34). The exploratory four-group comparison also remained statistically non-significant, F(3,56) = 0.860, *p* = 0.467, partial η² = 0.044. Mean haemoglobin decline in the sensitivity analysis was 1.64 ± 1.20 g/dL in the clipping group, 1.24 ± 0.93 g/dL in the shoelace-suture group, 1.42 ± 0.99 g/dL in the coagulation group, and 1.11 ± 0.44 g/dL in the no-occlusion group. Although the sensitivity analysis produced a larger pooled difference and effect estimate than the prespecified 24-hour analysis, neither the pooled nor four-group comparison reached statistical significance. A detailed analysis is presented in Supplementary Table S1.

### Sensitivity analyses

Sensitivity analyses were performed to assess whether the exploratory four-group difference in estimated blood loss was influenced by baseline imbalances. Fibroid count and largest-fibroid diameter were both positively associated with EBL on Spearman correlation analysis (fibroid count: ρ = 0.466, *p* < 0.001; largest-fibroid diameter: ρ = 0.459, *p* < 0.001).

Because EBL was right-skewed and strictly positive, ANCOVA on log-transformed EBL was used as the principal adjusted model. Conventional ANCOVA on raw EBL and Quade’s rank-based ANCOVA were performed as sensitivity analyses. Models progressively adjusted for age, baseline haemoglobin, fibroid count, and largest-fibroid diameter (Table 5).

**Table 5:** Sensitivity analyses of the exploratory four-group comparison of estimated blood loss Panel A: Progressive covariate-adjusted models

Adjustment model	Method	F statistic	df	*p*-value	Partial η²
**Unadjusted (Kruskal–Wallis)**	Kruskal–Wallis	H = 10.998	3	0.012^*^	ε² = 0.186
**Age only**	log-ANCOVA	F = 3.861	3, 55	0.014^*^	0.174
	Parametric ANCOVA	F = 3.457	3, 55	0.022^*^	0.159
	Quade’s rank ANCOVA	F = 4.204	3, 55	0.010^*^	0.187
**Age + baseline Hb**	log-ANCOVA	F = 3.770	3, 54	0.016^*^	0.173
	Parametric ANCOVA	F = 3.395	3, 54	0.024^*^	0.159
	Quade’s rank ANCOVA	F = 4.041	3, 54	0.012^*^	0.183
**Fibroid count + largest fibroid diameter**	log-ANCOVA	F = 3.076	3, 54	0.035^*^	0.146
	Parametric ANCOVA	F = 1.979	3, 54	0.128	0.099
	Quade’s rank ANCOVA	F = 2.907	3, 54	0.043^*^	0.139
**Age + fibroid count + largest fibroid**	log-ANCOVA	F = 2.962	3, 53	0.040*	0.144
	Parametric ANCOVA	F = 1.828	3, 53	0.153	0.094
	Quade’s rank ANCOVA	F = 2.830	3, 53	0.047^*^	0.138
**Age + baseline Hb + fibroid count + largest fibroid (Full model)**	log-ANCOVA	F = 2.916	3, 52	0.043^*^	0.144
	Parametric ANCOVA	F = 1.818	3, 52	0.155	0.095
	Quade’s rank ANCOVA	F = 2.741	3, 52	0.053	0.137

Sensitivity analyses assessed whether the exploratory four-group difference in estimated blood loss was influenced by baseline imbalances, particularly fibroid count and largest-fibroid diameter. Because EBL was right-skewed, log-ANCOVA was used as the principal adjusted model; Quade’s rank-based ANCOVA and conventional (parametric) ANCOVA on raw EBL were performed as sensitivity analyses. Effect sizes are reported as ε² for the unadjusted Kruskal–Wallis test and partial η² for the adjusted models. EBL, estimated blood loss; Hb, haemoglobin; * = statistically significant at *p* < 0.05

The exploratory four-group difference in EBL remained statistically significant after adjustment for age alone under all three methods: log-ANCOVA, F(3,55) = 3.861, *p* = 0.014, partial η² = 0.174; conventional ANCOVA on raw EBL, F(3,55) = 3.457, *p* = 0.022, partial η² = 0.159; and Quade’s rank-based ANCOVA, F(3,55) = 4.204, *p* = 0.010, partial η² = 0.187. Similar findings were observed after adjustment for age and baseline haemoglobin. Haemoglobin decline remained non-significant after adjustment for age alone (*p* = 0.547) and after adjustment for age and baseline haemoglobin (*p* = 0.540) (Table 6).

**Table 6:** Adjusted geometric mean EBL and geometric mean ratios from the fully adjusted log-ANCOVA model

Group	*n*	Adjusted geometric mean EBL, mL	95% CI (mL)	Geometric mean ratio versus no occlusion, (95% CI)	*p*-value
**Clipping**	15	256	205–318	0.94 (0.68–1.31)	0.733
**Shoelace**	15	268	219–327	0.99 (0.73–1.34)	0.942
**Coagulation**	16	192	159–232	0.71 (0.53–0.94)	0.022
**No occlusion (reference)**	14	271	217–338	Reference	—

Adjusted geometric means were estimated from the fully adjusted ANCOVA model applied to log-transformed EBL and were back-transformed to the original mL scale. The model included treatment group, age, baseline haemoglobin, fibroid count, and largest-fibroid diameter. Geometric mean ratios were calculated relative to the no-occlusion group; ratios below 1.00 indicate lower adjusted EBL. Pairwise comparisons were exploratory and were not adjusted for multiple testing. The estimates should therefore be interpreted cautiously in view of the small group sizes, baseline fibroid imbalance, residual confounding, and multiplicity of comparisons. *EBL* estimated blood loss, *Hb* haemoglobin, *CI* confidence interval. Pairwise comparisons were exploratory, derived post hoc from the fully adjusted model, and were not adjusted for multiple testing

After adjustment for fibroid count and largest-fibroid diameter, the results varied according to the analytical method. In the fully adjusted model incorporating age, baseline haemoglobin, fibroid count, and largest-fibroid diameter, the overall group effect remained statistically significant in the log-ANCOVA, F(3,52) = 2.916, *p* = 0.043, partial η² = 0.144. In contrast, the group effect was not statistically significant in the conventional ANCOVA on raw EBL, F(3,52) = 1.818, *p* = 0.155, partial η² = 0.095, or in Quade’s rank-based ANCOVA, F(3,52) = 2.741, *p* = 0.053, partial η² = 0.137.

These technique-specific pairwise estimates were derived from a post-hoc adjusted model, were exploratory, and were not adjusted for multiple comparisons; accordingly, they should not be interpreted as confirmatory evidence of superiority of any individual occlusion technique.

In the fully adjusted log-ANCOVA, the adjusted geometric mean EBL was 256 mL (95% CI 205–318) in the clipping group, 268 mL (95% CI 219–327) in the shoelace-suture group, 192 mL (95% CI 159–232) in the coagulation group, and 271 mL (95% CI 217–338) in the no-occlusion group. Relative to no occlusion, the adjusted geometric mean ratios were 0.94 (95% CI 0.68–1.31) for clipping, 0.99 (95% CI 0.73–1.34) for shoelace suture, and 0.71 (95% CI 0.53–0.94) for coagulation. These pairwise comparisons were exploratory and were not adjusted for multiple testing.

Overall, adjustment for fibroid burden attenuated the four-group association and produced method-dependent findings. The technique-specific estimates should therefore be considered exploratory and potentially influenced by residual confounding and multiplicity rather than interpreted as definitive evidence of superiority of any individual occlusion technique.

### Secondary outcomes: operative time and ovarian reserve

Secondary outcome data are presented in Table 7. Total operative time differed significantly across groups (*p* = 0.009). The no-occlusion group had the shortest operative time (median 120 min, IQR 110–132.5), while the clipping and shoelace groups required approximately 50–60 min longer (median 180 and 170 min, respectively). Thus, the median operative time was approximately 50–60 min longer in these two groups than in the no-occlusion group.

**Table 7:** Secondary outcomes: operative time and ovarian reserve

Outcome	Clipping (*n* = 15)	Shoelace (*n* = 15)	Coagulation (*n* = 16)	No Occlusion (*n* = 14)	*p*-value	Effect estimate
Operative time						
**Total operative time (min)**,** median (Q1–Q3)**	180 (120–220)	170 (120–230)	130 (110–180)	120 (110–132.5)	0.009	—
**Occlusion time (min)**,** median (Q1–Q3)** ^*^	28 (25–35)	35 (30–40)	20 (16.25–28.75)	—	0.002	ε² = 0.267
**Ovarian reserve: AMH**						
**Preoperative AMH (ng/mL)**,** median (Q1–Q3)**	2.12 (1.44–3.8)	3.1 (1.2–3.9)	1.58 (1.12–2.79)	1.26 (0.88–1.69)	0.018	—
**Postoperative AMH at 3 months (ng/mL)**,** median (Q1–Q3)**	2.04 (1.14–3.78)	3.02 (1.12–3.78)	1.35 (1.00–2.60)	1.07 (0.85–1.57)	0.120	—
**Adjusted group effect on post-op AMH** ^**†**^	—	—	—	—	0.385	F(3,55) = 1.035; partial η² = 0.053
**Within-group p-value** ^**‡**^	< 0.001	< 0.001	< 0.001	< 0.001	—	—
**Ovarian reserve: AFC**						
**Preoperative AFC**,** median (Q1–Q3)**	16 (10–28)	20 (10–26)	14 (12–17.75)	11 (6.75–15)	0.039	—
**Postoperative AFC at 3 months**,** median (Q1–Q3)**	14 (8–25)	20 (10–24)	13 (9–16)	9 (5.75–12.5)	0.017	—
**Adjusted group effect on post-op AFC** ^**†**^	—	—	—	—	0.799	F(3,55) = 0.337; partial η² = 0.018
**Within-group p-value** ^**‡**^	< 0.001	< 0.001	< 0.001	< 0.001	—	—

Data are presented as median (Q1–Q3), unless otherwise specified. This table presents secondary exploratory four-group comparisons. Total operative time and occlusion time were compared using the Kruskal–Wallis test. Occlusion time was assessed only among the three uterine artery occlusion groups. Postoperative AMH and AFC were analysed using ANCOVA or rank-based ANCOVA, as appropriate, with adjustment for the corresponding baseline value and age. Within-group preoperative-to-postoperative changes were assessed using the Wilcoxon signed-rank test. Unadjusted postoperative p values are presented descriptively and should be interpreted in the context of the significant baseline imbalances in AMH and AFC. The adjusted analyses did not identify detectable short-term between-group differences; however, these findings do not demonstrate equivalence or definitive ovarian safety. AMH, anti-Müllerian hormone; AFC, antral follicle count; ANCOVA, analysis of covariance

*Occlusion time was applicable only to the clipping, shoelace-suture, and coagulation groups

†Adjusted for the corresponding baseline ovarian reserve marker and age

‡ Within-group comparison of preoperative and three-month postoperative values

Among the three uterine artery occlusion methods, occlusion time also differed significantly (H [2] = 12.014, *p* = 0.002; ε² = 0.267). The shortest occlusion time was observed in the coagulation group (median 20 min, IQR 16.25–28.75), whereas the longest was observed in the shoelace group (median 35 min, IQR 30–40). The ε² estimate is reported descriptively because these technique-specific comparisons were exploratory.

Preoperative AMH and AFC differed significantly across groups (*p* = 0.018 and *p* = 0.039, respectively), as reported in Table 1. After adjustment for the corresponding baseline value and age, no detectable between-group differences were observed in postoperative AMH or AFC at three months (AMH: F[3,55] = 1.035, *p* = 0.385, partial η² = 0.053; AFC: F[3,55] = 0.337, *p* = 0.799, partial η² = 0.018).

All four groups demonstrated statistically significant within-group declines in AMH and AFC from preoperative to three-month postoperative assessment (all *p* < 0.001). However, the magnitude of these changes did not differ significantly among groups. Median ΔAMH ranged from − 0.10 to − 0.14 ng/mL (Kruskal–Wallis H [3] = 1.912, *p* = 0.590; ε² = 0.032), while median ΔAFC ranged from − 1 to − 2 follicles (H [3] = 1.138, *p* = 0.770; ε² = 0.019). Overall, no detectable short-term between-group difference in postoperative ovarian reserve markers was identified after adjustment for baseline imbalances. These findings should not be interpreted as evidence of equivalence or definitive ovarian safety.

### Safety and postoperative outcomes

Safety outcomes are presented in Table 8. Two intraoperative hemorrhagic complications related to uterine artery dissection occurred: one in the clipping group (6.7%) and one in the coagulation group (6.3%). No such events occurred in the shoelace-suture or no-occlusion groups (Fisher’s exact test, *p* = 1.000). No conversions to laparotomy or hysterectomy were required in any group. Intraoperative technique modification was required in two cases in the shoelace group (13.3%) and one case in the coagulation group (6.3%), compared with none in the clipping or no-occlusion groups; this difference was not statistically significant (*p* = 0.500).

All postoperative complications were classified as minor according to the Clavien–Dindo classification system. Postoperative ileus occurred in seven participants (11.7%) and was managed conservatively as a Grade I complication. Blood transfusion was required in 11 participants (18.3%) and was classified as a Grade II complication. No Grade III, IV, or V complications were observed.

Postoperative ileus occurred most frequently in the clipping group (4/15, 26.7%) compared with none in the no-occlusion group, although this difference was not statistically significant (Fisher’s exact test, *p* = 0.150). Hospital stay duration differed statistically across groups (Kruskal–Wallis H [3] = 7.880, *p* = 0.049; ε² = 0.134), although the clinical relevance of this difference is limited because the median length of stay was identical at 1 day in all groups.

**Table 8:** Safety and postoperative outcomes

Outcome	Clipping (*n* = 15)	Shoelace (*n* = 15)	Coagulation (*n* = 16)	No Occlusion (*n* = 14)	*p*-value
Intraoperative safety					
**Any intraoperative complication**,** n (%)**	1 (6.7%)	0	1 (6.3%)	0	1.000
**Conversion to laparotomy**,** n (%)**	0	0	0	0	
**Conversion to hysterectomy**,** n (%)**	0	0	0	0	
**Technique changed**,** n (%)**	0	2 (13.3%)	1 (6.3%)	0	0.500
**Postoperative complications by Clavien–Dindo grade**					
**Grade I (ileus)**,** n (%)**	4 (26.7%)	1 (6.7%)	2 (12.5%)	0	0.150
**Grade II (blood transfusion)**,** n (%)**	5 (33.3%)	3 (20%)	3 (18.8%)	0	0.140
**Grade III–V**,** n (%)**	0	0	0	0	—
**Hospital stay**					
**Hospital stay (days)**,** median (Q1–Q3)**	1 (1–2)	1 (1–2)	1 (1–2)	1 (1–1)	0.049

Data are presented as n (%) or median (Q1–Q3), as appropriate. Categorical outcomes were compared using Fisher’s exact test. Hospital stay was compared using the Kruskal–Wallis test. Conversion to laparotomy and conversion to hysterectomy were not statistically compared because no events occurred. Postoperative complications were graded according to the Clavien–Dindo classification. Grade II complications included blood transfusion. Transfusion criteria were Hb < 8 g/dL, Hb 8–9 g/dL with clinical signs of haemodynamic instability, or perioperative Hb decline > 3 g/dL. *Hb*  haemoglobin

## Discussion

This randomized trial was designed primarily to determine whether uterine artery occlusion, performed using different techniques, reduces intraoperative blood loss and 24-hour haemoglobin decline compared with conventional laparoscopic myomectomy without occlusion. Secondary exploratory objectives were to compare the feasibility, safety, operative outcomes, and short-term ovarian reserve effects of three uterine artery occlusion techniques: temporary clipping, shoelace suture, and permanent bipolar coagulation.

The clinical relevance of blood-loss reduction strategies during laparoscopic myomectomy is emphasized by the evidence-based practice guideline from the American Association of Gynecologic Laparoscopists (AAGL) [14], which supports several measures to minimize bleeding, including preoperative GnRH agonist therapy, intraoperative pharmacological agents such as misoprostol, epinephrine, vasopressin, and oxytocin, and surgical techniques including uterine artery occlusion. However, the guideline also highlights the limited availability of direct comparative data between different haemostatic approaches and occlusion techniques.

Although numerous studies have evaluated uterine artery occlusion as a strategy to reduce blood loss during laparoscopic myomectomy, the literature is largely limited to comparisons between a single occlusion technique and conventional laparoscopic myomectomy, with considerable heterogeneity in study design, surgical technique, and outcome reporting. Direct comparisons between multiple occlusion techniques within the same study population remain scarce. In this context, the present study provides a structured exploratory comparison of three distinct uterine artery occlusion techniques within a standardized surgical setting, while retaining the prespecified primary comparison of uterine artery occlusion versus no occlusion.

The primary pooled comparison did not detect a reduction in estimated blood loss with uterine artery occlusion. Although the observed group medians differed by 55 mL in the direction of higher EBL with occlusion, the Hodges–Lehmann estimate of the between-group location difference was 30 mL, with a 95% confidence interval ranging from − 50 to 110 mL. Thus, the result does not establish benefit, harm, or equivalence. Interpretation is further limited by the substantially greater-than-anticipated variability in EBL, which reduced the approximate power to detect the prespecified 50-mL difference to approximately 24%.

Transfusion requirements were numerically higher in the occlusion group (23.9% vs. 0%; risk difference 23.9% points, 95% CI 0.2–37.9; Fisher’s exact *p* = 0.053). The analysis showed no significant difference in 24-hour haemoglobin decline between uterine artery occlusion and no occlusion. However, the scheduled 24-hour haemoglobin value represented a post-transfusion measurement in the 11 participants transfused before this assessment, potentially attenuating the observed decline. A sensitivity analysis replacing these values with haemoglobin concentrations measured immediately before transfusion increased the pooled difference from 0.06 to 0.32 g/dL and the standardized effect estimate from Cohen’s d = 0.07 to 0.34, although the difference remained statistically non-significant. These findings suggest that the original 24-hour analysis may have underestimated perioperative haemoglobin decline among transfused participants, but the sensitivity analysis did not demonstrate a statistically significant between-group difference. Taken together, these findings do not establish a haemostatic benefit, harm, or absence of benefit from routine preparatory uterine artery occlusion. The limited power and imprecision of the primary comparison preclude definitive conclusions regarding whether occlusion should be used routinely or selectively.

In the secondary exploratory four-group analysis, estimated blood loss differed across surgical techniques, with median values ranging from 150 mL in the coagulation group to 360 mL in the clipping group (*p* = 0.012; ε² = 0.186). Post hoc analysis showed that the only pairwise comparison meeting the Bonferroni-adjusted threshold was coagulation versus clipping. No individual occlusion technique demonstrated superiority over the no-occlusion control. Importantly, the four-group finding was influenced by baseline fibroid burden. After adjustment for fibroid count and largest fibroid diameter, the group effect was attenuated and became method-dependent: the fully adjusted group effect remained statistically significant in the log-transformed ANCOVA but was not statistically significant in either conventional ANCOVA on raw EBL or Quade’s rank-based ANCOVA. Although the fully adjusted log-ANCOVA estimated a lower geometric mean EBL for coagulation than for no occlusion (geometric mean ratio 0.71, 95% CI 0.53–0.94), this pairwise comparison was exploratory and was not adjusted for multiple testing. These findings should therefore be interpreted as exploratory and hypothesis-generating rather than definitive evidence of technique superiority.

An important observation was that the no-occlusion group had lower median blood loss than both the clipping and shoelace suture groups. This does not necessarily indicate that avoidance of occlusion is superior in all cases, but it suggests that the net benefit of uterine artery occlusion may depend on the balance between haemostatic gain and the additional dissection required to achieve vascular control. Retroperitoneal dissection, vessel isolation, clip placement, or temporary suture application may themselves contribute to operative time and cumulative blood loss. Whether preparatory uterine artery occlusion provides greater benefit in selected patients at high anticipated bleeding risk warrants evaluation in adequately powered studies.

Baseline fibroid burden was a key factor in interpreting the exploratory four-group findings. The clipping group had a higher fibroid count than the other arms, and both fibroid count and largest fibroid diameter were significantly associated with EBL. Adjustment for these variables reduced the estimated group effect and made statistical significance dependent on the analytic method used. Therefore, the higher observed blood loss in the clipping group and the lower observed blood loss in the coagulation group should not be interpreted as clean causal effects of the occlusion method alone.

The biological rationale for uterine artery occlusion is supported by the vascular anatomy of fibroids. As described by Sinha et al. [15], uterine fibroids derive much of their vascular supply from the uterine arteries, whereas the surrounding myometrium benefits from collateral circulation via the utero-ovarian arcade. In principle, interruption of uterine arterial flow may reduce fibroid perfusion and facilitate myomectomy. The lower observed blood loss in the coagulation group may be consistent with immediate and definitive interruption of uterine arterial flow; however, this interpretation remains exploratory because superiority over no occlusion was not demonstrated and baseline fibroid burden differed between groups.

Previous randomized and observational studies have generally reported reductions in intraoperative blood loss with temporary or permanent uterine artery occlusion, although the magnitude of benefit has varied. Ji et al. [16] reported reduced blood loss with temporary occlusion without prolonging operative time, while Hiratsuka et al. [17] observed reduced bleeding at the expense of longer operative duration. Liu et al. [6] and Ciavattini et al. [11] also reported favorable haemostatic effects using transient ligation and bipolar coagulation, respectively. Meta-analytic evidence by Witkowiak et al. [18] demonstrated an overall reduction in blood loss, but with heterogeneity across studies. In contrast, the present trial did not show a significant benefit for uterine artery occlusion when the three occlusion techniques were considered together, raising the possibility that technique-specific factors may influence outcomes; however, this requires confirmation in adequately powered comparative trials.

Despite overall consistency in the literature, variability persists across studies. Dubuisson et al. [19] highlighted heterogeneity in study design and outcome assessment, particularly in methods used to estimate blood loss. In the present study, quantitative measurement using calibrated suction–irrigation and subtraction of the recorded irrigation volume provided a standardized estimate of intraoperative blood loss. Nevertheless, the measurement was recorded by operating-room personnel who were aware of the allocated surgical technique, and the possibility of differential measurement bias cannot be excluded.

An important and recurring observation across the literature is the discrepancy between estimated blood loss and haemoglobin decline. While several studies, including those by Vercellino et al. [20] and Balulescu et al. [21], emphasized haemoglobin decline as an indicator of perioperative blood loss, our study did not demonstrate significant differences in haemoglobin decline despite variation in estimated blood loss between individual techniques. This may reflect the multifactorial nature of perioperative haemodynamics, including intraoperative fluid administration, redistribution of intravascular volume, and the relatively modest absolute differences in blood loss between groups. In addition, the scheduled 24-hour postoperative haemoglobin value represented a post-transfusion measurement in the 11 participants transfused before that assessment. Pre-transfusion haemoglobin values were subsequently retrieved for all transfused participants and incorporated into a sensitivity analysis. Substitution of these values increased the pooled between-group difference in haemoglobin decline from 0.06 to 0.32 g/dL and Cohen’s d from 0.07 to 0.34, although the result remained statistically non-significant. The sensitivity analysis was limited by the non-uniform timing of haemoglobin assessment, because pre-transfusion measurements obtained 4–6 h postoperatively were used for transfused participants while the scheduled 24-hour value was retained for non-transfused participants. Similar findings have been reported in recent randomized trials, such as Moratalla-Bartolomé et al. [22], reinforcing the limited sensitivity of haemoglobin decline in detecting moderate differences in bleeding within minimally invasive surgery.

In contrast to the inconsistent haemostatic benefit, operative time was consistently longer with uterine artery occlusion in the prespecified primary comparison (*p* = 0.007). In the exploratory four-group analysis, operative time also differed across groups (*p* = 0.009), with the longest durations observed in the clipping and shoelace suture groups and the shortest durations in the no-occlusion and coagulation groups. The prolonged operative time associated with clipping and shoelace suture techniques may be attributed to their higher technical demands, including meticulous vascular dissection, accurate clip application with potential risk of slippage, and the additional steps required for temporary suture placement and subsequent reversal. In contrast, bipolar coagulation may require fewer device-management steps once the uterine artery has been isolated, which could partly explain the shorter operative time observed in that group.

No conversions to laparotomy or hysterectomy and no Grade III–V postoperative complications occurred in this trial. Intraoperative complications were rare, with only two events recorded across all groups, and the observed postoperative complications were Grade I or II. However, transfusion occurred in 11 participants in the occlusion groups and in none of the participants in the no-occlusion group, while postoperative ileus was also numerically more frequent in the clipping group. These numerical trends should be interpreted cautiously because the clipping group also had greater baseline fibroid burden and higher observed blood loss. These findings are consistent with previous reports, including those by Vercellino et al. [20] and Kim et al. [23]. Similarly, large cohort data from MacKoul et al. [24] support the safety of vascular control techniques, even in more complex surgical cases. Although no severe perioperative morbidity was observed, the sample size was insufficient to reliably compare uncommon complications or establish equivalent safety among techniques.

The potential impact of uterine artery occlusion on ovarian reserve is an important consideration. Randomized trials by Streuli et al. [25] and Hashemi et al. [26] demonstrated no significant changes in serum AMH levels following temporary or permanent occlusion techniques. In the present study, after adjustment for baseline ovarian reserve markers and age, no short-term between-group difference in postoperative AMH or AFC was detected. However, the study was not powered to establish equivalence or to exclude clinically meaningful long-term effects on ovarian reserve. In addition, the 3-month follow-up period limits interpretation of AMH recovery and future fertility outcomes. Therefore, the ovarian-reserve findings should be interpreted as absence of a detected short-term difference rather than evidence of definitive ovarian safety, particularly after permanent bipolar coagulation of the uterine arteries.

The absence of a detected short-term between-group difference in ovarian reserve markers may be partly explained by collateral circulation via the utero-ovarian arcade, which may help maintain ovarian perfusion despite interruption of uterine blood flow. However, this remains speculative in the absence of direct ovarian perfusion assessment. Moreover, the potential advantage of temporary techniques—preservation of uterine and ovarian perfusion for future fertility—remains unverified within the 3-month follow-up window. The theoretical benefit of reversible occlusion on endometrial receptivity and pregnancy outcomes warrants dedicated investigation in future longitudinal studies.

This study has several strengths. It used a randomized design and directly compared multiple uterine artery occlusion techniques within a standardized surgical setting, enabling a structured exploratory evaluation of their relative perioperative profiles. All procedures were performed by two experienced laparoscopic surgeons, thereby reducing operator-related variability. Estimated intraoperative blood loss was assessed quantitatively as the volume collected in calibrated suction canisters after subtraction of the recorded irrigation volume, providing a standardized assessment across groups. However, because the operating-room personnel responsible for recording the measurement were necessarily aware of treatment allocation, the possibility of measurement bias remains. The inclusion of operative, safety, and short-term ovarian reserve outcomes also provides a broad assessment of the clinical implications of these techniques.

Nonetheless, certain limitations should be acknowledged. The study focused on short-term perioperative outcomes and did not include long-term follow-up for fibroid recurrence, reproductive outcomes, endometrial receptivity, or longitudinal ovarian reserve recovery. Subtle performance biases related to surgeon preference, technical familiarity, or the ergonomic demands of specific occlusion methods cannot be entirely excluded. In addition, the study was conducted in specialized centers by experienced laparoscopic surgeons, which may limit generalizability to other clinical environments.

Although this study employed a four-arm randomized design, the sample size calculation was based on the prespecified primary comparison between uterine artery occlusion performed using different techniques and no occlusion. Consequently, the trial was not powered to detect modest differences among individual occlusion techniques or to support multiple definitive pairwise comparisons. In addition, baseline fibroid burden differed across groups, particularly with a higher fibroid count in the clipping group. Although adjusted sensitivity analyses were performed, residual confounding cannot be excluded. Therefore, the four-group analyses should be regarded as exploratory and hypothesis-generating. Future adequately powered multicenter trials with stratified or blocked randomization are warranted to confirm the comparative effectiveness of individual uterine artery occlusion techniques.

An important limitation concerns the assumptions underlying the sample-size calculation. The a priori calculation assumed an SD of 60 mL for estimated blood loss. In the observed data, the SD was 134.7 mL in the pooled uterine artery occlusion group and 101.7 mL in the no-occlusion group, giving a weighted within-group pooled SD of approximately 128.0 mL. Using the same two-sample framework as the original calculation, the achieved 46:14 allocation provided approximately 24% power to detect the prespecified 50-mL difference based on the observed variability, compared with approximately 77% under the original SD assumption. The primary comparison was therefore materially underpowered, and its non-significant result should not be interpreted as demonstrating absence of benefit or equivalence. The Hodges–Lehmann estimate was 30 mL in the direction of higher EBL with occlusion, but the 95% confidence interval ranged from a 50-mL reduction to a 110-mL increase, remaining compatible with both clinically important benefit and harm.

The retrospective public registration of this trial represents an important limitation. Although ethical approval was obtained and the original ethics-approved protocol—including the study design, eligibility criteria, four-arm randomization plan, interventions, specified outcomes, and follow-up schedule—was finalized before recruitment commenced, public registration occurred after enrollment had begun. Consequently, independent prospective verification of the prespecified protocol through the public registry was not possible. No changes were made to the eligibility criteria, interventions, or prespecified outcomes after recruitment started. Additional adjusted and sensitivity analyses undertaken to examine the robustness of the findings are identified transparently as such in the manuscript.

In addition, recruitment of one additional eligible participant after the pre-intervention withdrawal constituted a protocol deviation from the originally planned recruitment procedure, although the participant was randomized using the next sequentially numbered, opaque, sealed envelope from the original randomization sequence and allocation concealment was maintained.

Simple unrestricted randomization was used without stratification or blocking. Given the modest total sample size and four-group allocation, this approach carried a risk of baseline covariate imbalance, which was observed in age, ovarian reserve markers, and fibroid burden. The use of block randomization with stratification by clinically important predictors, such as age and fibroid burden, would have reduced this risk and should be considered in future studies.

In addition, estimated intraoperative blood loss—the primary outcome—was recorded intraoperatively by operating-room personnel who were necessarily aware of the allocated surgical technique. Although a standardized quantitative measurement method was used, the absence of blind assessment may have introduced measurement bias.

The scheduled 24-hour haemoglobin measurement was obtained after transfusion in 11 participants and was therefore influenced by the administered blood products. Although pre-transfusion haemoglobin values were retrieved for all transfused participants and incorporated into a sensitivity analysis, these measurements were obtained 4–6 h postoperatively, whereas the scheduled 24-hour value was retained for non-transfused participants. The resulting non-uniform timing limits direct comparability and means that the sensitivity analysis should be interpreted as supportive rather than as a replacement for the prespecified primary analysis.

## Conclusions

In this randomized trial, uterine artery occlusion performed using different techniques did not produce a detectable reduction in estimated intraoperative blood loss or 24-hour haemoglobin decline compared with no occlusion and was associated with longer operative time. However, the observed variability in blood loss substantially exceeded the a priori assumption, leaving the primary comparison underpowered and its confidence interval compatible with both clinically important benefit and harm.

Secondary exploratory analyses suggested differences among individual occlusion techniques, with permanent bipolar coagulation showing the lowest observed blood loss and the shortest operative time among the occlusion methods. Nevertheless, these findings were method-dependent, the study was not powered for definitive pairwise comparisons, baseline fibroid burden differed between groups, and superiority of any individual technique over no occlusion was not established.

No detectable short-term between-group difference in ovarian reserve markers was observed after adjustment for baseline imbalances. Larger, adequately powered trials are required to determine whether uterine artery occlusion provides clinically meaningful benefit and whether any individual occlusion technique is preferable.

## Supplementary Information

Supplementary Material 1.

Supplementary Material 2.

## Data Availability

The datasets generated and analyzed during the current study are available from the corresponding author on reasonable request.
