# Supplementary material for: Comparing different techniques of uterine artery occlusion during laparoscopic myomectomy: a randomized controlled trial
Source: BMC Womens Health. 2026 Aug 25;26:428. doi: 10.1186/s12905-026-04799-z (PMC13508237; doi:10.1186/s12905-026-04799-z)
Supplement: Supplementary file 2 [file 12905_2026_4799_MOESM2_ESM.docx]

**Supplementary Figure 1. Lateral (transperitoneal) approach to uterine artery identification during laparoscopic myomectomy.**

Laparoscopic view demonstrating the lateral pelvic anatomy following peritoneal incision. The round ligament is identified superiorly, with the infundibulopelvic ligament laterally. The external iliac artery is visualized coursing along the pelvic sidewall. Blunt dissection is performed to develop the pararectal/paravesical space, facilitating exposure of the uterine artery at its origin from the internal iliac artery.

**Supplementary Figure 2. Laparoscopic clipping of the uterine artery at its origin from the anterior division of the internal iliac artery.**

Intraoperative laparoscopic view demonstrating identification of the uterine artery in relation to the obliterated umbilical artery within the paravesical space. Following meticulous dissection and skeletonization, a vascular clip is applied to the uterine artery to achieve immediate mechanical occlusion.

**Supplementary Figure 3. Shoelace suture technique for uterine artery ligation during laparoscopic myomectomy.**

After skeletonization of the uterine artery, an absorbable suture (Vicryl 1-0 polyglactin 910) is passed around the vessel using a right-angle or dissecting instrument. The suture ends are then tightened progressively in an alternating manner, producing a shoelace-like constrictive effect that ensures uniform circumferential compression of the artery.

**Supplementary Figure 4. Laparoscopic identification and coagulation of the uterine artery**

Intraoperative laparoscopic views demonstrating precise identification of the uterine artery in close relation to the ureter (“water under the bridge”). The ureter is visualized coursing inferiorly and laterally, serving as a critical landmark to avoid thermal or mechanical injury. Blunt and sharp dissection are used to skeletonize the uterine artery, followed by controlled bipolar coagulation.

**Supplementary table S1. Sensitivity analysis: haemoglobin decline using pre-transfusion haemoglobin values for transfused participants**

| **Analysis** | **UAO using different techniques** | **No occlusion** | **Effect estimate/statistic** | **p-value** | **Effect size** |
| --- | --- | --- | --- | --- | --- |
| **Prespecified analysis using 24-hour postoperative Hb** | 1.17 ± 0.78 g/dL | 1.11 ± 0.44 g/dL | Mean difference 0.06 g/dL | 0.759* | d = 0.07 |
| **Sensitivity analysis using pre-transfusion Hb for transfused participants** | 1.43 ± 1.04 g/dL | 1.11 ± 0.44 g/dL | Mean difference 0.32 g/dL | 0.106* | d = 0.34 |
| **Four-group prespecified analysis (ANOVA)** | — | — | F [3,56] = 0.455 | 0.715 | η² = 0.025 |
| **Four-group sensitivity analysis (ANOVA)** | — | — | F [3,56] = 0.860 | 0.467 | η² = 0.044 |

*Values are mean ± SD. In the prespecified analysis, haemoglobin decline was calculated as preoperative haemoglobin minus the scheduled 24-hour postoperative haemoglobin value. For participants transfused before the 24-hour measurement, this represented a post-transfusion value. In the sensitivity analysis, the pre-transfusion haemoglobin value measured 4–6 hours postoperatively at the time of the transfusion decision was used for all 11 transfused participants; the scheduled 24-hour value was retained for non-transfused participants. The pooled intervention-versus-control comparison was performed using Welch’s t-test because of unequal variances between the two pooled groups. The exploratory four-group comparison was performed using one-way ANOVA. UAO, uterine artery occlusion; Hb, haemoglobin; SD, standard deviation. *Welch's t-test (unequal variances).*
